# Assigning a Prominent Role to “The Patient Experience” in Assessing the Quality of Integrated Care for Populations with Multiple Chronic Conditions

**DOI:** 10.5334/ijic.4656

**Published:** 2019-09-26

**Authors:** Mieke Rijken, Manon Lette, Caroline A. Baan, Simone R. de Bruin

**Affiliations:** 1 Nivel (Netherlands Institute for Health Services Research), Utrecht, NL; 2 RIVM (National Institute for Public Health and the Environment), Centre for Nutrition, Prevention and Health Services, Department of Quality of Care and Health Economics, Bilthoven, NL; 3 University of Eastern Finland, Faculty of Social Sciences and Business Studies, Department of Health and Social Management, Kuopio, FI; 4 EMGO+ Institute/Amsterdam Public Health Research Institute, Department of General Practice and Elderly Care Medicine, Amsterdam UMC, Amsterdam, NL; 5 Tranzo (Scientific Center for Care and Welfare), TS Social and Behavioral Sciences, Tilburg University, Tilburg, NL

**Keywords:** integrated care, quality assessment, patient experience, user involvement, multimorbidity, frailty

## Abstract

In response to growing populations of citizens with multiple chronic conditions, integrated care models are being implemented in many countries. Based on our experiences from three EU co-funded actions (ICARE4EU, SUSTAIN, JA-CHRODIS), we notice that users’ experiences are not always taken into account when assessing the quality of integrated care, whereas research shows that it is in this particular domain that quality improvement is most evident.

The greatest value of integrated care for people with multiple chronic conditions may not lie in its potential to improve their health or reduce their use of services, but in its potential to improve their care experience, by strengthening person-centred decision-making and delivering care and support accordingly. Collaborations of care providers, (representatives of) people with multiple chronic conditions and researchers need to develop appropriate methods and measures to include users’ experiences in quality assessment of integrated care.

## Context and aim

Driven by the rapid increase of the numbers of inhabitants with multiple chronic conditions, countries are currently reforming their health and care systems to respond better to the comprehensive needs of these people. This is reflected in the implementation of integrated care at national or local level for (older) people with multiple chronic conditions [1,2]. As a consequence, health system performance and quality assessment needs to be adapted to monitor and evaluate these developments. Many countries today collect data on users’ experiences with health services, in addition to data about access, service utilization, health outcomes and costs. However, assessing users’ experiences of integrated care has been limited, as the applied measures almost all focus on disease-specific interventions or single encounters with care professionals, which are unable to capture the concept of integrated care [3,4]. Moreover, they do not do justice to the concept of person-centeredness [4,5], which should be the basis of care for people with multiple chronic conditions, as the goals of care need to be set in close interaction with these persons based on their individual values and priorities [6,7], and care delivery should be organised accordingly.

With this paper, we aim to call special attention to including “the patient experience” when assessing the quality of care for people with multiple chronic conditions, as we believe that this particular Triple Aim-component [8] has not been sufficiently valued on its own merit. We reflect upon its value and assessment challenges from our experience as consortium leaders of two large-scale EU funded projects, ICARE4EU (www.icare4eu.org) and SUSTAIN (www.sustain-eu.org) (see **Box 1**), and our involvement in the EU Joint Action CHRODIS (www.chrodis.eu).

## The value of assessing the patient experience

**Figure 1:**
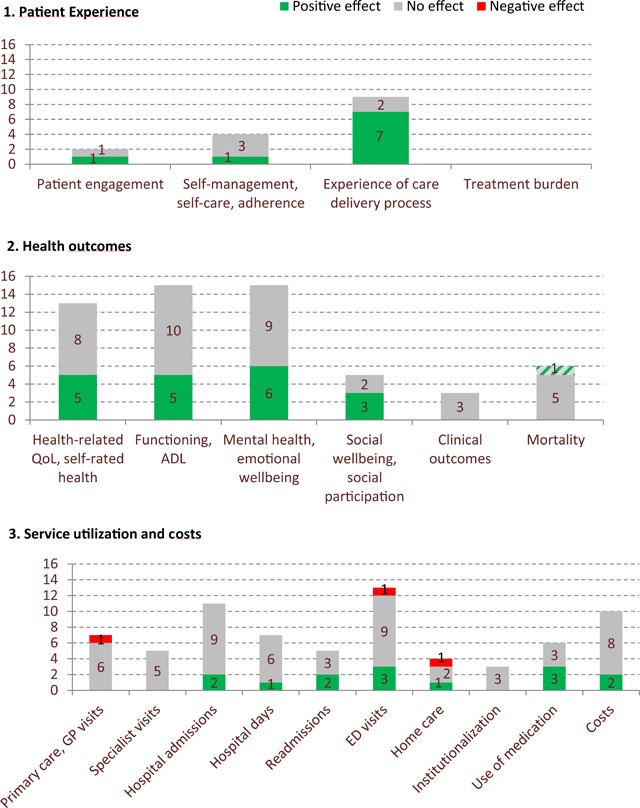
Effects of integrated care programmes for multi-morbidity or frailty management on Triple Aim-components (N = 29 studies).

As reflected in many international and national quality assessment frameworks [9], person-centredness is considered a key element of high-quality care, and its crucial role in quality assessment of care for people with multiple chronic conditions has recently been emphasized based on a broad scoping review [10]. It speaks for itself that this requires these people or their representatives to be involved in quality assessment [3]. The importance of including “the patient experience” when evaluating integrated care for people with multi-morbidity or frailty is illustrated in Figure 1, which summarizes the results of four systematic reviews [11,12,13,14]. We reconsidered the studies included in these reviews and excluded those that evaluated integrated care initiatives for persons with a specific chronic condition or a specific combination of chronic conditions. Rearranging the results of the 29 included studies according to the Triple Aim framework, reveals that users’ experience of the care delivery process was not assessed in the majority of the studies. Studies in which it was assessed reported predominantly more positive experiences as a result of implementing (elements of) integrated care. This suggests that people with multi-morbidity or frailty experience better care as a result of interventions to strengthen person-centeredness or integration of care, while beneficial effects on health outcomes and service utilization/costs are often absent.

**Box 1: The ICARE4EU project and the SUSTAIN project**

ICARE4EU

The project *Innovating care for people with multiple chronic conditions in Europe* (ICARE4EU) run from 2013 until 2016, and was co-funded by the Health Programme of the European Commission. It aimed to identify and analyse innovative care models targeting multi-morbidity in European countries. Data were collected in 2014/2015 with the help of country experts in 31 countries, who identified and reported on integrated care practices that had a focus on multi-morbidity in their country. Eligible practices were contacted to provide detailed information by means of an online questionnaire. In this way, data were received from 101 practices in 25 countries. Site visits were made to eight practices to obtain a more in-depth understanding of their characteristics and results. Observations and recommendations for policy and practice have been published in country factsheets, case reports, an overview “state-of-the-art” report, scientific papers and policy briefs (all available from www.icare4eu.org).

SUSTAIN

The project *Sustainable Tailored Integrated Care for Older People in Europe* (SUSTAIN) run from 2015 until 2019, and was co-funded under Horizon 2020, the Framework Programme for Research and Innovation (2014–2020) from the European Commission (EC). SUSTAIN’s objectives were twofold: 1. to support and monitor improvements of integrated care initiatives for older people living at home with multiple health and social needs, and in so doing move towards more person-centred, prevention-oriented, safe and efficient care; and 2. to contribute to the adoption and application of these improvements to other health and care systems, and regions in Europe. The SUSTAIN-project was carried out by thirteen partners from eight European countries: Austria, Belgium, Estonia, Germany, Norway, Spain, the Netherlands, and the United Kingdom. With the exception of Belgium, all countries selected two integrated care initiatives in their country for being supported by the SUSTAIN project to improve their current way of working (www.sustain-eu.org).

The lack of evidence in the outcome and cost domains may relate to weaknesses of the studies, such as a lack of power or a limited follow-up period. But a recent high-quality study [15] among 1546 patients with multi-morbidity that aimed to improve continuity, coordination and efficiency of care neither showed an effect on health (related) outcomes (EQ-5D-5L [16], self-rated health, HADS [17]), whereas the number of consultations with general practitioners and nurses slightly increased. However, what this study clearly demonstrated were, once again, improvements in patients’ experiences and satisfaction.

Without denying that methodological shortcomings of the studies could have contributed to the absence of effects on health outcomes, service use and costs, we wish to add the possibility that integrated care may not be able to substantially improve such outcomes among populations with multiple chronic conditions. Due to the complexity of their health problems, a large part of the consultations with healthcare providers, medication use and hospital admissions cannot be avoided and many health outcomes are difficult to improve. This is not to say that no outcomes should be assessed; outcomes that are highly valued by people with multiple chronic conditions, e.g., social wellbeing or participation, deserve more attention as well (see Figure 1). As Berwick and colleagues state, the Triple Aim components should be balanced carefully [8]. It is up to policy-makers and stakeholders to discuss the weight of each of the components, but in doing this, it is essential to have a thorough understanding of the target population and its needs. When assessing the quality of integrated care for people with multiple chronic conditions, attaching more value to users’ experiences should be considered.

## The challenge of assessing the patient experience

Having said that, based on our experiences and as reflected in Figure 1, we conclude that assessing the experiences of people with multimorbidity or frailty is not common practice yet. In the ICARE4EU project, it was shown that in only half of the 101 integrated care programmes for people with multi-morbidity, users’ perceptions of the quality of care or their level of satisfaction were included as quality indicators [18].

Why is it that the experiences of users with multiple chronic conditions are not included more often? Firstly, it may be that care providers are not encouraged to do so, as users’ experiences are still not considered a key indicator of countries’ health system performance [19]. Second, there may be (perceived) barriers. Managers of some integrated care programmes involved in the ICARE4EU project reported legal restrictions related to privacy protection or the strict rules of Research Ethics Committees (RECs) in their country. They stated it was complicated and time-consuming to involve patients or clients in quality assessment. Some even indicated that it was not allowed in their country and they feared to break the rules. Besides, there were professionals who believed they should not bother high-need patients with interviews or surveys, as this would imply an additional burden to these people. We recognize that it is of utmost importance to minimize measurement burden for these persons. We also agree that preparing a research file for medical ethical review takes time. However, since all information materials, the data collection protocol and instruments will be available for implementation right after the preparatory process, it may save time in the end. The European Network of Research Ethics Committees (www.eurecnet.org) provides access to training materials and EU and national legislation to support local RECs, which could also be helpful for professionals and researchers of studies involving human participants [20]. Many national RECs also provide such support.

The integrated care initiatives participating in the SUSTAIN project were supported by the SUSTAIN consortium to improve their care processes. In these initiatives much effort was put on assessing the perceptions of the older service users. It was decided to focus on aspects that were considered most appropriate for vulnerable older people, such as their experiences with care coordination and the extent to which they experienced control over the care they received. Nevertheless, it appeared difficult to assess these aspects due to a lack of valid measuring instruments and doubts about the ability of structured questionnaires to capture quality aspects that truly reflect older people’s values.

The development of patient-reported experience measures (PREMs) has really taken off these days [19], but many are not appropriate to assess the quality of integrated care because of their limited scope: they focus on care provided by a single discipline or on medical interventions for a specific condition. PREMs for quality assessment of integrated care for people with multiple chronic conditions should go beyond disease-specific interventions and single encounters [4,21]. Moreover, they should cover aspects of person-centred care such as care coordination and alignment to patients’ goals. Some PREMs do meet a number of these criteria, for instance the PACIC [22] or PAIEC [23]. The P3CEQ [5,24] specifically focuses on the evaluation of aspects of person-centred coordinated care for older users. The latter questionnaire has now been translated in Dutch, Estonian, German, Norwegian and Spanish as an activity of the SUSTAIN project. The appropriateness of the P3CEQ to reflect the experience of older vulnerable people in the quality improvement initiatives supported by the SUSTAIN project is currently being studied.

## Conclusions and recommendations

Integrated care for people with multiple chronic conditions should be valued on its true merits. Its greatest value may not lie in its potential to improve health outcomes or reduce service utilization and costs, but in its potential to improve the care delivery process for people with high needs for care and support; i.e., assuring that their voice is heard in every phase of the care process, that their goals and priorities are the guiding principles of the individual care plan, and that care and support is delivered accordingly. This asks for quality indicators and assessment instruments that capture elements of person-centred integrated care that are highly valued by people with multiple chronic conditions. Collaborations of care providers, (representatives of) people with multiple chronic conditions and researchers within and across countries could facilitate the development and implementation of appropriate methods and measures. Furthermore, as innovations to improve care for (older) people with multiple chronic conditions do not make sense without knowing whether these people experience better care, developing and implementing indicators to assess users’ experience of person-centred integrated care should go hand-in-hand with care innovation.
